# AI in Health Care Service Quality: Systematic Review

**DOI:** 10.2196/69209

**Published:** 2025-11-05

**Authors:** Eman Alghareeb, Najla Aljehani

**Affiliations:** 1 Eastern Health Cluster, Saudi Electronic University, Abi Bakr As Siddiq Road, P.O. Box 93499, Riyadh, 11673, Kingdom of Saudi Arabia, ‪+966 50 549 1908‬; 2 Department of Public Health, College of Health Sciences, Saudi Electronic University, Riyadh, Kingdom of Saudi Arabia

**Keywords:** artificial intelligence, AI, health care quality, Saudi Arabia, technology

## Abstract

**Background:**

Artificial intelligence (AI) is a rapidly evolving technology with the potential to revolutionize the health care industry. In Saudi Arabia, the health care sector has adopted AI technologies over the past decade to enhance service efficiency and quality, aligning with the country’s technological thrust under the Saudi Vision 2030 program.

**Objective:**

This review aims to systematically examine the impact of AI on health care quality in Saudi Arabian hospitals.

**Methods:**

A meticulous and comprehensive systematic literature review was undertaken to identify studies investigating AI’s impact on health care in Saudi Arabia. We collected several studies from selected databases, including PubMed, Google Scholar, and Saudi Digital Library. The search terms used were “Artificial Intelligence,” “health care,” “health care quality,” “AI in Saudi Arabia,” “AI in health care,” and “health care providers.” The review focused on studies published in the past 10 years, ensuring the inclusion of the most recent and relevant research on the effects of AI on Saudi Arabian health care organizations. The review included quantitative and qualitative analyses, providing a robust and comprehensive understanding of the topic.

**Results:**

A systematic review of 12 studies explored AI’s influence on health care services in Saudi Arabia, highlighting notable advancements in diagnostic accuracy, patient management, and operational efficiency. AI-driven models demonstrate high precision in disease prediction and early diagnosis, while machine learning optimizes telehealth, electronic health record compliance, and workflow efficiency, despite adoption challenges like connectivity limitations. Additionally, AI strengthens data security, reduces costs, and facilitates personalized treatment, ultimately enhancing health care delivery.

**Conclusions:**

The review underscores that AI technologies have significantly improved diagnostic accuracy, patient management, and operational efficiency in Saudi Arabia’s health care system. However, challenges such as data privacy, algorithmic bias, and robust regulations require attention to ensure successful AI integration in health care.

## Introduction

The health care industry is on the cusp of a significant transformation driven by the integration of artificial intelligence (AI). The fusion of big data, cloud computing, artificial neural networks, and machine learning has led to the development of machines that mimic human intelligence [1]. This study focuses on these machines referred to as AI, which can perceive, recognize, learn, react, and problem-solve, and explores their potential to revolutionize health care. The introduction of these innovative technologies is a possibility and a reality in health care, promising a bright future. In Saudi Arabia, the integration of AI in the health care industry has become increasingly prevalent over the last decade. For example, a study by Al Kuwaiti et al [1] states that AI applications have revolutionized health care, mainly since the advent of COVID-19, and presents a promising solution to future health care needs. Further, the Ministry of Health uses eHealth as a transformative tool to improve health care delivery through digital and information technology, aligning with the Saudi Vision 2030 implementation [2] and underscoring the significant and essential benefits of technology, particularly AI, in health care, wherein technology is transforming the diagnostic process into a more collaborative and efficient one [3]. This benefits health care organizations, professionals, patients, their families, researchers, and decision makers. The potential of AI in health care is not just promising but also instilling a strong sense of confidence in its capabilities.

Deep learning models have emerged as a game-changer in the health care industry, especially in the use of the Internet of Things (IoT) in health care. Shah and Chircu [4] highlighted that these models are being used with remarkable accuracy to monitor patients’ heart failure, leveraging the power of intelligent IoT-based frameworks. This approach has proven highly effective in preventing heart failure–related complications and reducing the burden on health care providers, as well as in enhancing patient outcomes, decreasing hospital readmissions, and lowering health care costs [4]. Integrating deep learning models in the IoT in health care framework has revolutionized the health care industry and potentially transformed health care services, with the ability to classify patients as alive or deceased and share health records with medical professionals for emergency assistance [5].

Furthermore, the AI is revolutionizing early diagnoses and predictions of dental implant cases, thereby significantly enhancing health care services in dentistry [6]. AI algorithms can also analyze medical images such as X-rays and magnetic resonance imaging, thereby aiding radiologists in detecting and diagnosing medical conditions, which leads to earlier detection and better patient treatment outcomes [7].

Almalawi et al [7] predicted that AI was expected to be indispensable in supporting clinical and supplementary applications that lead to more intelligent and efficient operations and care in the future. By using secure and interoperable data, health care organizations, health systems, and health plan companies can leverage the power of AI to gain valuable insights and drive informed decision-making. Businesses embracing AI will likely experience significant benefits, including short-term cost savings and long-term competitive advantages. Also, by adopting AI-powered solutions, these organizations can enhance their services, improve customer engagement, and stay ahead of the curve in a constantly evolving industry [7]. With AI technology at their fingertips, health care organizations can unlock new opportunities for growth and success while providing their customers with the best possible experience [7].

Indeed, as noted in the study by Almalawi et al [7], AI has the potential to transform the health care industry, but there are also potential pitfalls to consider. First, with the advancements in AI technology, there are legitimate concerns about its impact on society, particularly privacy, security, and employment. Privacy concerns are valid as AI systems can collect and use personal information, such as medical records, financial data, and location information. Establishing robust data protection protocols is imperative to prevent data breaches and the misuse of personal information. Additionally, AI systems can perpetuate biases and discrimination if the data they were trained on are biased or reflect existing inequalities. Therefore, unbiased data are necessary for training AI systems; addressing these concerns can ensure a more informed and prepared approach to integrating AI in health care [7]. Second, AI can pose a security risk if not designed and implemented securely. Malicious actors can exploit AI systems, leading to unauthorized data access or other harmful consequences. Health care privacy laws, such as those in Saudi Arabia, can also present challenges for legal regulations, allowing physicians to disclose patient information for educational purposes [7]. Finally, there are concerns about AI’s impact on employment, with predictions suggesting that it may displace workers in industries like manufacturing and transportation. However, AI primarily uses supervised machine learning, and its role in Saudi Arabia’s health care policy needs further clarification. To integrate AI effectively in health care, it is crucial to set clear goals and define specific objectives for AI integration, such as improving diagnostic accuracy, optimizing resource allocation, and enhancing patient management. Develop comprehensive policies and create guidelines that address ethical considerations, data privacy, and security measures to ensure responsible AI use.

Effective AI integration in health care requires a structured approach encompassing stakeholder engagement, pilot program implementation, and professional training. Engaging health care providers, policymakers, and patients ensures that AI policies align with practical and ethical considerations [8]. Implementing pilot programs facilitates real-world testing, allowing for evidence-based refinements and the expansion of successful initiatives [9,10]. Additionally, comprehensive training programs equip health care professionals with the necessary skills to seamlessly integrate AI into clinical practice, thereby enhancing patient care and operational efficiency [11,12]. Collectively, these measures support responsible AI adoption in health care, contributing to improved diagnostic accuracy, operational efficiency, and patient outcomes [7,12]. These actions will maximize AI’s potential in health care [7]. Additionally, it is essential to note that AI could create new job categories and enhance human abilities, potentially leading to a more diverse and dynamic workforce [7].

Integrating AI technology in health care has become increasingly important for improving the quality and efficiency of health care services, especially in Saudi Arabian hospitals [7,8]. However, there is a lack of information available in the existing literature regarding the impact of AI on health care implementation in Saudi Arabia. Previous research has mainly focused on clinical outcomes, with less emphasis on the impact of AI on health care providers and the efficiency of adapting to AI in health care settings [3,5,6]. To address this gap, this study aims to examine how AI affects the quality of health care services in Saudi Arabia. By consolidating existing evidence, this study aims to provide valuable insights for policymakers and inform health care professionals and administrators about the most effective ways to optimize AI applications to improve efficiency and enhance the quality of patient care. The study reviewed the accuracy and reliability of AI systems compared to human doctors and the potential cost savings and increased efficiency resulting from their use. Ultimately, this research might support Saudi Arabia in making informed decisions about leveraging AI to improve health care services while prioritizing health care providers’ and patients’ welfare and privacy.

This study focuses exclusively on Saudi Arabia to provide a contextually relevant analysis of AI integration within its health care sector. Given the nation’s digital transformation under the Saudi Vision 2030 program, the centralized nature of Saudi Arabia’s health care system allows for a targeted evaluation that accounts for unique regulatory frameworks, cultural perceptions, and health care challenges, distinguishing it from other countries. By synthesizing evidence on AI’s role in diagnostic accuracy, patient management, and operational efficiency, the study supports evidence-based decision-making and offers insights for policymakers and health care professionals seeking to optimize AI-driven health care solutions. The aim of this study is to systematically review the available evidence to answer the following research question: What is the impact of AI on the quality of health care services in Saudi Arabia?

The significance of this review lies in its focus on a rapidly expanding area of health care transformation. AI adoption has the potential to elevate care quality, improve patient outcomes, reduce costs, and enhance efficiency. By consolidating the available evidence within the Saudi Arabian context, this study provides a timely and contextually relevant understanding of AI’s contributions and challenges. The findings aim to guide health care leaders in selecting appropriate AI applications, shaping workforce training, and designing governance frameworks that ensure responsible and sustainable integration of AI in clinical practice.

## Methods

### Study Design and Search Strategy

We conducted a systematic search of the literature in accordance with the PRISMA (Preferred Reporting Items for Systematic Reviews and Meta-Analyses) 2020 guidelines (Checklist 1). Five electronic databases—PubMed or MEDLINE, Web of Science, Scopus, Google Scholar, and the Saudi Digital Library—were searched to identify studies evaluating the impact of AI on health care services in Saudi Arabia. The search combined controlled vocabulary terms (eg, Medical Subject Headings) and free-text keywords related to AI and health care. The following concepts were applied: “artificial intelligence” OR “machine learning” OR “deep learning” AND “healthcare” OR “health services” OR “healthcare quality” OR “diagnostic accuracy” AND “Saudi Arabia” OR “Saudi healthcare.” Additional terms were included to capture provider-level perspectives, such as “healthcare providers” OR “clinical decision support.”

The search covered the period from January 2014 to April 2024 to ensure contemporary relevance, reflecting the accelerated adoption of AI in Saudi health care during the past decade. No language restrictions were applied at the search stage; however, only English language full texts were included at the screening stage. Boolean operators (AND and OR) and database-specific filters were used to refine results. The reference lists of all included studies were also hand-searched to identify additional relevant publications.

### Criteria

#### Inclusion Criteria

Studies were included if they were original empirical investigations—such as observational studies (cross-sectional, cohort, or case control), quasiexperimental studies, case series, or surveys—conducted in Saudi Arabia between January 2014 and April 2024. Eligible studies needed to explicitly evaluate the use of AI in health care delivery, with a focus on diagnostic accuracy, patient management, or operational efficiency. Both quantitative and qualitative designs were considered, and studies were required to involve health care providers (eg, physicians, nurses, pharmacists, and allied health professionals) and patients receiving health care services in Saudi Arabian hospitals or clinics, whether governmental or private. Only studies published in English were included.

#### Exclusion Criteria

The exclusion criteria were applied to remove studies that did not meet the scope of this review. These included reviews (systematic, narrative, or scoping); editorials; conference abstracts without full data; and opinion pieces. Studies were also excluded if they were conducted outside Saudi Arabia; if the study population did not include health care providers or patients (eg, students, policymakers, or the general public); or if the focus was not directly related to AI in health care. Studies published before 2014 were excluded to ensure that the review reflects contemporary developments in AI adoption under the Saudi Vision 2030 program.

### Data Extraction and Quality Assessment

This study is based on original research and academic papers from journals. The Mendeley References Manager software (Elsevier) identified all the records from the database to display the collected data [13]*.* The data were then entered into a Microsoft Excel worksheet to locate all the studies. After that, all titles were screened on the basis of the inclusion and exclusion criteria. Abstracts of the articles were analyzed in depth to ensure the quality and relevance of the academic literature included in the review process. Full-text screening was then done, and all these steps were presented on the PRISMA flow diagram. The systematic review followed the widely accepted PRISMA 2020 guidelines, which serve as a comprehensive flow diagram to ensure transparency and reproducibility [14]. The PRISMA flow diagram visually represents the study selection process from initial identification to the final inclusion of studies in the review.

To ensure the quality of the review, each included research paper was evaluated carefully at a later stage through the Joanna Briggs Institute (JBI) critical appraisal checklist [15]. The JBI critical appraisal checklist enhances systematic reviews by ensuring methodological rigor, assessing bias, and improving the reliability of included studies. It supports transparent and reproducible research, strengthening evidence-based health care decisions. The methodological quality was assessed in the studies chosen using the JBI methodology’s tailored criteria for different kinds of research. We performed the critical appraisal of eligible analytical cross-sectional studies for 8 studies, the critical appraisal of eligible cohort study research for 3 studies, and the critical appraisal of quasiexperimental studies for 1 study.

## Results

### Study Selection

**Figure 1.**
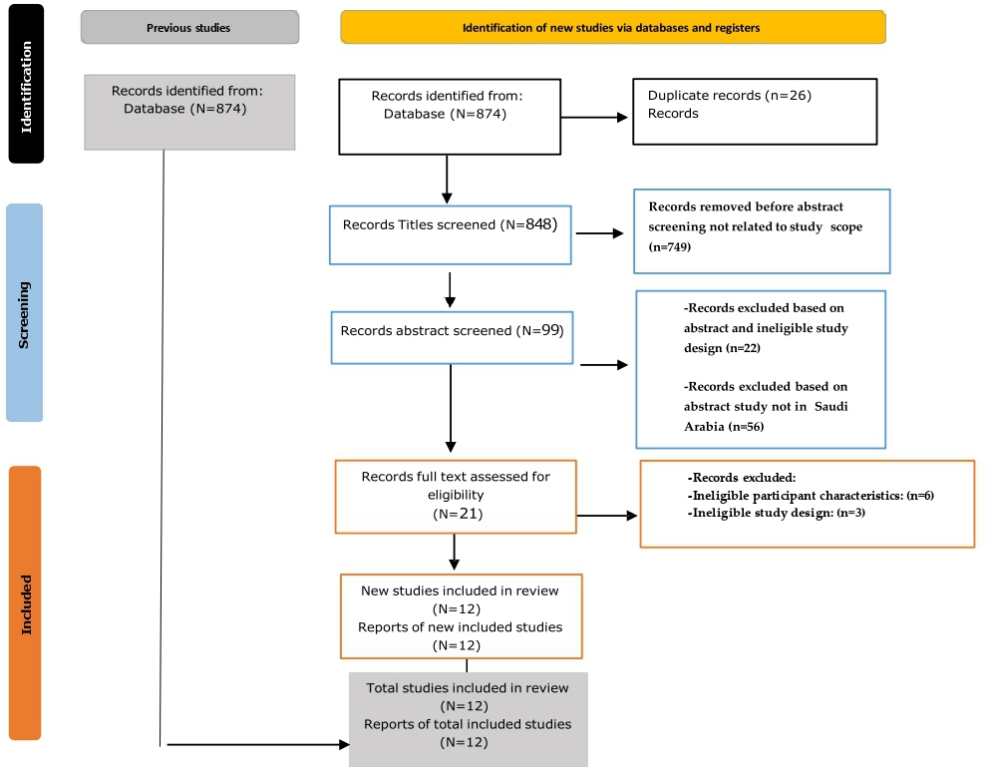
PRISMA (Preferred Reporting Items for Systematic Reviews and Meta-Analyses) flow diagram.

At the beginning of the review process, 874 records were identified across 3 databases: 295 from Google Scholar, 293 from PubMed, and 286 from the Saudi Digital Library (Table 1). Of the total, 26 duplicates were removed, leaving 848 unique records. Following title screening, 748 records were excluded for irrelevance and 99 abstracts were reviewed. Of these, 22 were excluded due to ineligible study design and 56 because they were not conducted in Saudi Arabia; 22 full texts were assessed, of which 9 were excluded (6 for ineligible populations and 3 for design). A final total of 12 studies were included. The PRISMA flow diagram is provided in Figure 1.

**Table 1.** Database sources: Boolean operators^a^ were used to run the searches between March 2024 and April 2024.

Database sources	Records, n
Google Scholar	295
PubMed	293
Saudi Digital Library databases	286

a The study aimed to investigate the impact of artificial intelligence on health care organizations in Saudi Arabia, drawing from existing literature. The search strategy involved the use of specific search phrases such as “Artificial Intelligence,” “healthcare,” “health quality,” “(AI) in Saudi Arabia,” and “(AI) in healthcare.” Boolean operators, including AND and OR, were strategically used to combine and exclude these search phrases, thereby refining the search results to be more precise.

### Study Themes and Distribution

The included studies addressed 4 major themes: diagnostic accuracy (n=3), patient management (n=3), operational efficiency (n=3), and quantified impact (n=3; Table 2). These themes capture the breadth of AI applications in Saudi health care and highlight the diverse ways AI has been studied, from early diagnostic models to workforce efficiency and provider perceptions (MultimediaAppendices 1  2).

**Table 2.** Study themes and distribution.

Theme	Studies, n	Study examples
Diagnostic accuracy	3	Aljameel et al [16], Ibrahim et al [17], Olatunji et al [18]
Patient management	3	Alghamdi et al [11], Al Yousef et al [12], Olatunji et al [18]
Operational efficiency	3	Alharbi and Almutiq [6], Al Habib and Alharthi [9], Seliaman et al [19]
Quantified impact	3	Almalawi et al [7], Serbaya et al [20], Aldossari [21]

### Diagnostic Accuracy

Three studies demonstrated that AI can improve diagnostic precision in Saudi health care contexts by leveraging clinical, imaging, and laboratory data. In the context of the COVID-19 pandemic, Aljameel et al [16] applied a random forest classifier to records from King Fahad University Hospital and achieved 95% accuracy and an area under the curve (AUC) of 0.99, suggesting that AI could serve as an effective early-warning tool for predicting disease severity and outcomes. This has direct implications for triaging patients in high-demand hospital settings. In oncology, Ibrahim et al [17] developed the AMAN detection system for interpreting equivocal mammograms, reporting 87% image classification accuracy, a 95% AUC, and 92% balanced accuracy, thereby confirming the feasibility of cost-effective deep learning–based systems to support breast cancer screening. In neurological care, a study tested 4 machine learning algorithms on hospital-based clinical and laboratory records, with support vector machines achieving 95.6% accuracy and an area under the receiver operating characteristic curve of 0.97 [22]. These results highlight the potential of AI to detect Alzheimer disease at earlier stages, where interventions may have a greater impact. Collectively, these studies show how AI can be integrated across diverse diagnostic pathways in Saudi Arabia, from infectious disease to oncology and neurology.

### Patient Management

Three studies assessed how AI supports personalized care and treatment planning. Olatunji et al analyzed data from 569 patients with multiple sclerosis using explainable AI, a method that not only produced robust predictions but also provided insight into the decision-making process of the algorithms. This interpretability allowed clinicians to better understand the rationale for predictions, strengthening trust and adoption in clinical settings. Alahmadi et al [23] developed a gradient boosting model using records of 622 patients with COVID-19, achieving 96.3% accuracy and identifying 23 predictive features, including comorbidities and beta-blocker use, which may reduce disease severity. This work underscores the value of AI for risk stratification and treatment prioritization during public health emergencies. Al Yousef et al [12] applied data mining techniques to electronic health records from 5 National Guard Health Affairs hospitals to predict diabetes onset. While the models performed acceptably, missing data limited accuracy; however, the study demonstrated the feasibility of using routinely collected hospital data for early disease prediction in Saudi Arabia. These examples illustrate the expanding role of AI in chronic disease management and highlight its potential for precision medicine approaches.

### Operational Efficiency

Three studies investigated AI applications for workflow optimization and organizational performance. Al Habib and Alharthi [9] used machine learning to predict physician noncompliance with electronic health record documentation. The random forest model achieved an AUC of 0.891, an *F*_1_-score of 0.831, and a recall of 0.831, demonstrating strong predictive performance and offering a practical solution for hospitals to proactively address compliance issues. Seliaman evaluated physician and pharmacist perceptions of clinical decision support systems (BESTCare 2.0), which are widely implemented in the National Guard hospitals. Findings indicated that usability, availability of patient information, and accessibility were critical factors influencing acceptance, highlighting both the potential of clinical decision support systems and the challenges in integrating them seamlessly into clinical workflows. In dentistry, Alharbi and Almutiq [6] explored AI models for predicting dental implant cases, showing that AI could improve treatment planning and reduce complications, thereby streamlining dental services. Together, these studies suggest that AI has important applications not only in clinical care but also in enhancing hospital efficiency and administrative oversight.

### Quantified Impact and Adoption

Three studies measured the broader impact of AI adoption in health care delivery. Alghamdi et al [11] reported that 47% of health care providers surveyed had used telehealth applications, but barriers such as poor internet connectivity, lack of time, and insufficient training limited widespread use. Serbaya et al [20] conducted a survey of health care workers in Jeddah and found that while 75% understood AI principles, only 50% were willing to use AI in medical decision-making. This gap highlights cultural and trust challenges that could slow AI adoption, even in settings where technical capability exists. Aldossari [21] showed that younger physicians, particularly those aged <30 years, expressed higher satisfaction with telehealth and demonstrated greater openness to expanded AI use in their practice. These findings emphasize the need for targeted strategies to improve provider engagement, training, and trust in AI tools.

### Critical Appraisal

Critical appraisal indicated moderate to high quality across most included studies. Cross-sectional studies scored between 50% and 100% across appraisal items, while cohort and quasiexperimental designs achieved scores ranging from 27% to 72%. Habib and Alharthi [9] achieved the highest appraisal score (100%), reflecting rigorous methodology, comprehensive outcome measures, and strong predictive performance. Although variability in quality was observed, particularly among quasiexperimental studies, the majority demonstrated methodological soundness and provided valuable insights into the role of AI in Saudi health care (Tables3  4). Only one quasiexperimental study (Ibrahim et al [17]) met the inclusion criteria. The study satisfied 4 of 9 quality appraisal items (44%), with clear reporting for participant selection and outcome measurement but limited information regarding confounding control and intervention description.

**Table 3.** Critical appraisal of an eligible analytical cross-sectional study.

Score	Q8	Q7	Q6	Q5	Q4	Q3	Q2	Q1	References
75%	Yes^a^	Yes	Unclear^b^	Unclear	Yes	Yes	Yes	Yes	Aljameel et al [16]
75%	Yes	Yes	Unclear	Unclear	Yes	Yes	Yes	Yes	Alghamdi et al [11]
100%	Yes	Yes	Yes	Yes	Yes	Yes	Yes	Yes	Al Habib and Alharthi [9]
75%	Yes	Yes	Unclear	Unclear	Yes	Yes	Yes	Yes	Seliaman M et al [19]
50%	Yes	Yes	Unclear	Unclear	Yes	Unclear	Yes	Unclear	Olatunji et al [24]
75%	Yes	Yes	Unclear	Unclear	Yes	Yes	Yes	Yes	Aldossari [21]
62.5%	Yes	Yes	Unclear	Unclear	Yes	Unclear	Yes	Yes	Alahmadi et al [23]
75%	Yes	Yes	N/A^c^	Unclear	Yes	Yes	Yes	Yes	Serbaya et al [20]

a Criterion clearly satisfied.

b Criterion uncertain or partially reported.

c N/A: not applicable.

**Table 4.** Critical appraisal of an eligible cohort study.

Score	Q11	Q10	Q9	Q8	Q7	Q6	Q5	Q4	Q3	Q2	Q1	References
72%	Yes^a^	N/A^b^	N/A	N/A	Yes	Yes	Yes	Yes	Yes	Yes	Yes	Olatunji et al [18]
27%	Yes	N/A	N/A	N/A	Yes	N/A	Unclear^c^	Unclear	N/A	N/A	Yes	Olatunji et al [25]
45%	Yes	N/A	N/A	N/A	Yes	Yes	Unclear	Yes	Unclear	Unclear	Yes	Yousef et al [12]

a Criterion clearly satisfied.

b N/A: not applicable.

c Criterion uncertain or partially reported.

## Discussion

### Principal Findings

This systematic review identified 12 eligible studies that examined the impact of AI on health care services in Saudi Arabia. The main findings show that AI applications have primarily focused on 4 domains: diagnostic accuracy, patient management, operational efficiency, and quantified impact. Across these studies, AI consistently demonstrated potential to improve diagnostic precision, support clinical decision-making, streamline health care workflows, and enhance cost-effectiveness. The integration of AI into health care represents a transformative development with profound implications worldwide, including in Saudi Arabia. The establishment of the Saudi Data and Artificial Intelligence Authority in August 2019 by royal decree marked a significant milestone in advancing the nation’s digital transformation under [2,8]. AI’s capacity to enhance diagnostic accuracy, streamline health care processes, and reduce operational costs is well documented. Johnson et al [10] examined the impact of AI on the health care sector in the United States, highlighting several advantages that align with findings observed in Saudi Arabia.

### Diagnostic Accuracy

AI-driven models have demonstrated significant advancements in medical diagnostics, enabling more precise disease identification and improving patient outcomes. For instance, Aljameel et al [16] developed a machine learning model for predicting COVID-19 outcomes, achieving 95% accuracy with an AUC of 0.99, thereby demonstrating AI’s potential in pandemic response. Similarly, Ibrahim et al [17] introduced the AMAN system for breast cancer detection, reporting an 87% accuracy in mammography and a 95% AUC, reinforcing AI’s role in oncological diagnostics. These findings align with global efforts to enhance diagnostic precision through AI-driven methodologies.

### Patient Management

AI has been instrumental in advancing patient management by facilitating early disease detection and optimizing treatment strategies. Olatunji et al [18] developed a machine learning model for early multiple sclerosis diagnosis, allowing for timely intervention and personalized treatment, which is critical for managing chronic conditions. Alghamdi et al [11] examined telehealth adoption among health care providers, revealing that 47% of surveyed professionals used telehealth, although barriers such as limited time availability and limited connectivity impeded broader implementation [26-28]. These insights underscore AI’s role in enhancing health care accessibility and personalized care delivery.

### Operational Efficiency

Beyond clinical applications, AI improves operational workflows and compliance monitoring. Al Habib and Alharthi [9] applied machine learning techniques to predict electronic health record documentation compliance, achieving an AUC score of 0.891, thereby assisting in identifying noncompliant physicians and supporting regulatory adherence. Furthermore, Alharbi and Almutiq explored AI applications in predicting dental implant cases, demonstrating AI’s ability to enhance patient outcomes and reduce complications associated with dental procedures [3]. These advancements underscore AI’s role in improving the efficiency of health care systems.

### Quantified Impact

AI integration extends to health care data security, cost reduction, and competitive advantages. Almalawi et al [7] emphasized the importance of secure health care data management, highlighting AI’s potential in mitigating cybersecurity risks while improving efficiency in health care administration. Similarly, Al Yousef et al [12] developed predictive models for early diabetes diagnosis, aiding in proactive disease prevention and effective health monitoring despite persistent technical challenges. AI-driven predictive analytics can significantly reduce health care costs and contribute to data-driven decision-making models.

### Regulatory and Cultural Considerations

Despite these advancements, notable differences exist between Saudi Arabia and the United States regarding data privacy and security concerns. While both countries acknowledge the importance of data protection, their regulatory frameworks diverge significantly. Saudi Arabia aligns AI integration with National Data Governance Regulations, fostering a centralized approach to data protection. In contrast, the United States operates within a multifaceted regulatory landscape that includes the Health Insurance Portability and Accountability Act (HIPAA) alongside various federal and state privacy laws, resulting in a more fragmented approach to data governance [29].

Resistance to AI adoption among health care providers has also been observed across both regions. In Saudi Arabia, skepticism is often linked to concerns about the potential erosion of the human element in patient care and the technological adaptations required for AI integration [8]. Conversely, in the United States, reluctance is more commonly associated with apprehensions regarding job displacement and challenges in integrating AI with existing health care IT infrastructure [10].

### Trust and Adoption Among Health Care Professionals

Several factors, including professional roles and cultural attitudes, influence AI adoption. A study by Serbaya et al [20] found that pharmacists exhibited a higher level of trust in AI compared to physicians and nurses, a trend also observed in European health care settings. Pharmacists’ familiarity with medication management technologies and their perception of AI-assisted decision-making as objective may contribute to this pattern. Additionally, gender disparities in AI confidence levels have been documented, with male health care professionals displaying greater confidence in AI applications than their female counterparts. Findings from previous studies attribute this discrepancy to greater exposure to technology among male professionals, job roles requiring technological engagement, and societal norms influencing interactions with emerging technologies [13,30,31]. Addressing these disparities through targeted educational programs and inclusive AI development strategies is crucial for equitable technology adoption.

### Recommendations

The following are actionable recommendations for stakeholders:

1. Enhancing data privacy and security
2. Implement robust data protection protocols
3. Develop clear data governance policies to safeguard patient information [2]
4. Address algorithmic bias
5. Ensure the availability of diverse and representative data
6. Conduct regular bias audits to maintain fairness in AI algorithms [3]

The following are recommendations to improve infrastructure and training:

1. Invest in technological infrastructure
2. Provide continuous training for health care professionals to use AI technologies effectively [2]

The following are recommendations to foster collaboration and innovation:

1. Encourage public-private partnerships
2. Create innovation hubs to drive advancements in AI solutions for health care [1]

The following are regulatory suggestions for AI implementation:

- Establishing regulatory frameworks
  1. Develop comprehensive AI regulations
  2. Implement regulatory sandboxes to safely deploy AI technologies [1]
- Ensuring transparency and accountability
  1. Mandate transparency in AI systems
  2. Establish accountability mechanisms to foster trust and address adverse outcomes [2]

### Challenges in AI Adoption in Saudi Health Care

The following are challenges with regard to data quality and availability:

1. Ensure high-quality data are available for AI training
2. Establish standards for data collection, storage, and processing that align with global best practices, as delineated by Health Level Seven International [32] and the Global Alliance for Genomics and Health [33]

The following are challenges with regard to regulations and ethical guidelines:

1. Formulate comprehensive regulations for ethical AI use in health care
2. Integrate ethical principles (fairness, transparency, and accountability) into AI system development. The Saudi government may consider frameworks such as the European Union’s guidelines on AI ethics, which emphasize respect for human autonomy, harm prevention, promotion of fairness, and the need for explicability [32].
3. Impose strict penalties for patient privacy or data security violations, including unauthorized data transmissions and data mining activities conducted without explicit patient consent
4. Secure informed consent from patients before using their data. Align data protection policies with the principles of the General Data Protection Regulation [8].

The following are challenges faced in providing training for medical personnel:

1. Provide adequate training for health care professionals to use AI tools effectively
2. Emphasize continuous education in AI to enhance the understanding of AI technologies, data privacy, and bias mitigation. A study conducted by the American Medical Association highlighted the need for ongoing education and training for health care professionals in AI to ensure they are adequately equipped to care for patients and enhance patient care [8].

### Conclusions

Integrating AI into health care policy and enhancing the continuous use of AI health devices requires a multifaceted approach. By defining clear objectives, involving stakeholders, and focusing on user-friendly design and constant monitoring, we can ensure that AI technologies are effectively used to improve health care outcomes. Addressing challenges such as data privacy, infrastructure investments, and bias mitigation will be crucial for successful implementation.

AI can potentially revolutionize health care in Saudi Arabia by predicting patient outcomes; personalizing medicine; streamlining administrative tasks; and improving accuracy, efficiency, and cost-effectiveness. However, it is critical to also focus on proper training for medical personnel while maintaining data privacy and addressing biases in AI algorithms. The Saudi Arabian health care sector can benefit from AI in many ways, including predicting disease outbreaks, enhancing patient admissions processes, and supporting ongoing education for medical professionals. Furthermore, deploying telehealth services and other digital health strategies highlights AI’s vital role in the region’s health care future. To fully realize AI’s potential in health care, it is essential to tackle the associated challenges and risks. With careful implementation and training, AI can lead to more accurate and efficient health care services, improving patient outcomes and reducing costs.

## Supplementary material

10.2196/69209 Multimedia Appendix 1 Themes of artificial intelligence applications in health care studies.

10.2196/69209 Multimedia Appendix 2 Number of publications by author and year.

10.2196/69209 Checklist 1 PRISMA checklist.

## Abbreviations

AI: artificial intelligence
AUC: area under the curve
HIPAA: Health Insurance Portability and Accountability Act
IoT: Internet of Things
JBI: Joanna Briggs Institute
PRISMA: Preferred Reporting Items for Systematic Reviews and Meta-Analyses
