# Supplementary figures and images for: AI in Health Care Service Quality: Systematic Review
Source: JMIR AI. 2025 Nov 5;4:e69209. doi: 10.2196/69209 (PMC12594439; doi:10.2196/69209)

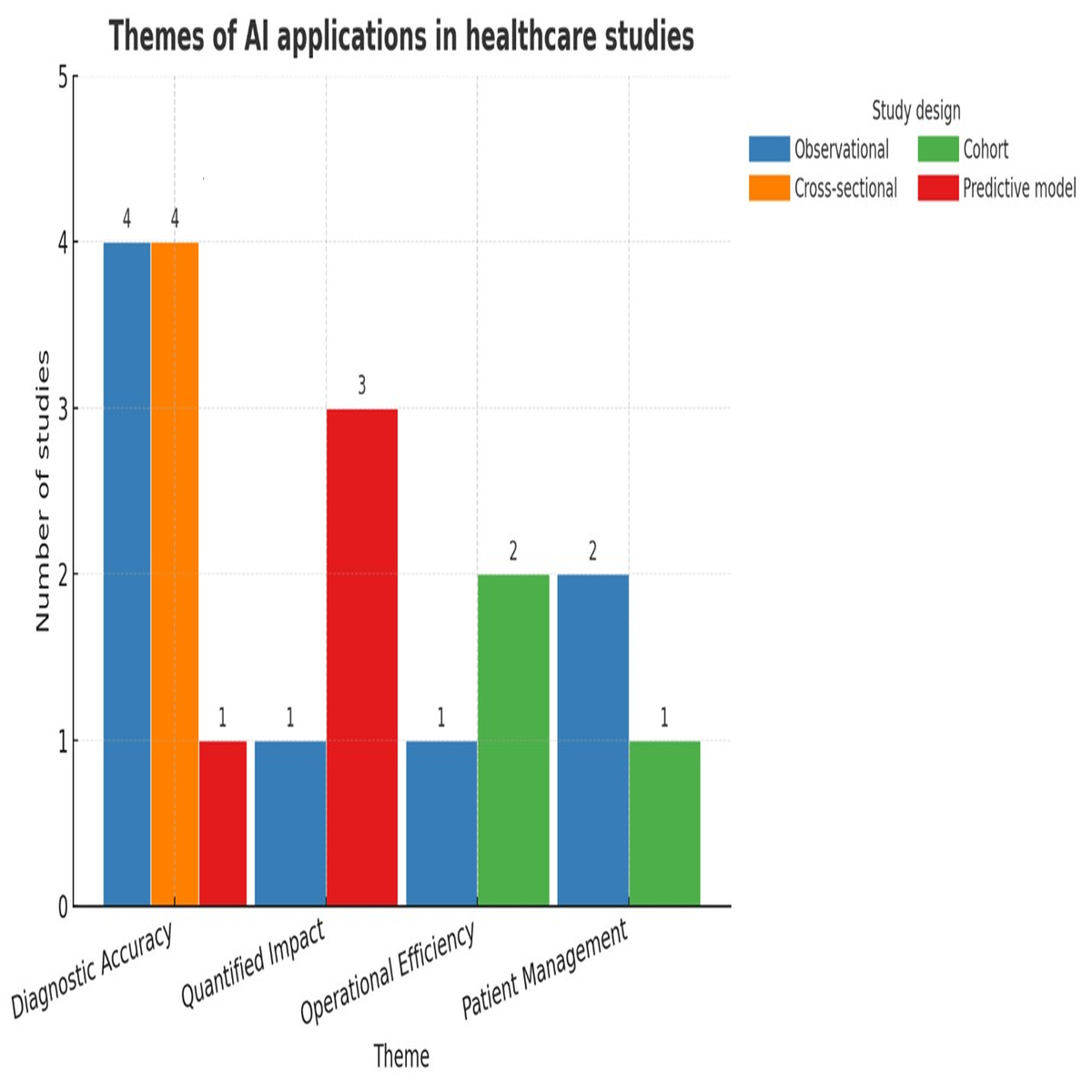

Supplement: Multimedia Appendix 1 [file ai-v4-e69209-s001.png]

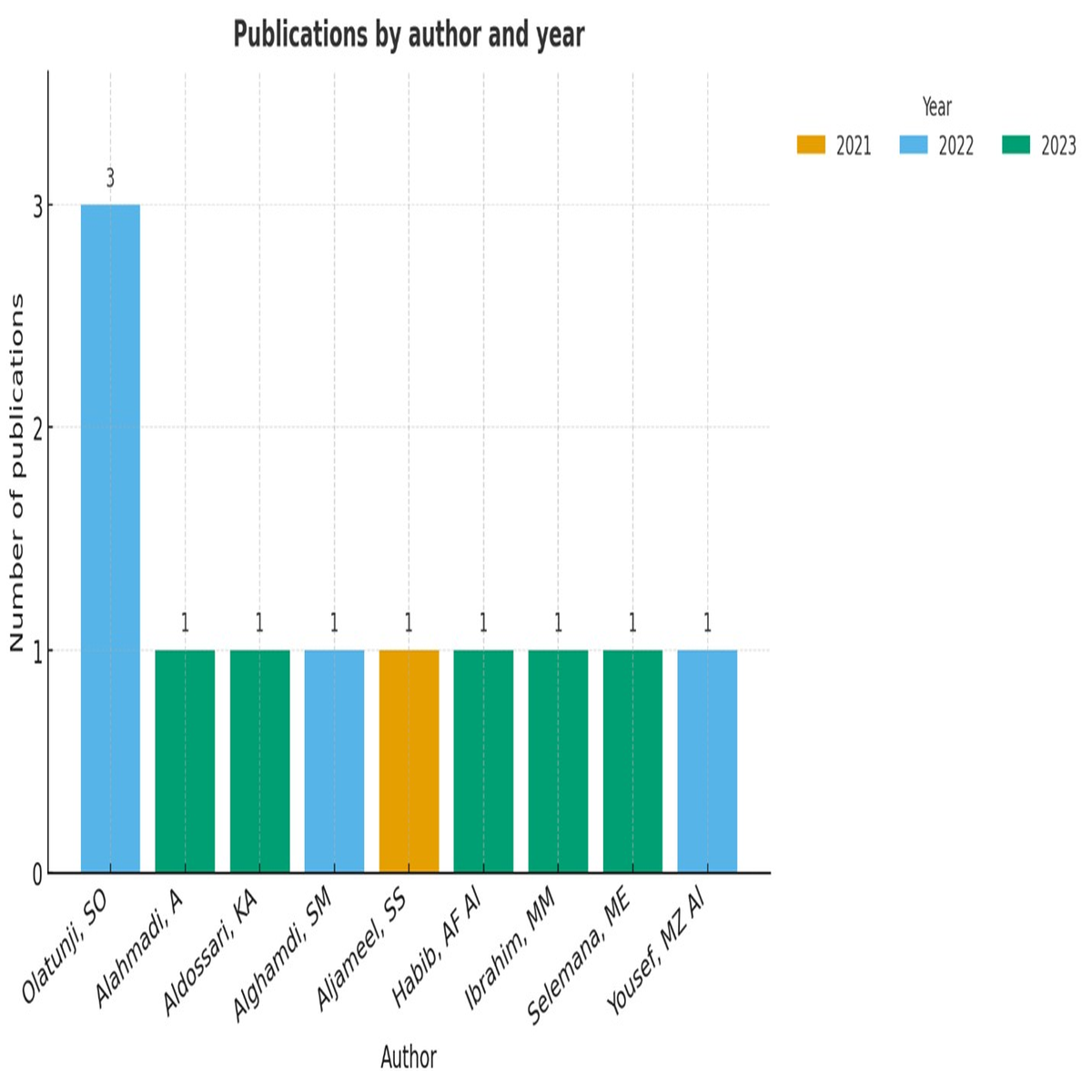

Supplement: Multimedia Appendix 2 [file ai-v4-e69209-s002.png]
